# Publisher Correction: Structure of the membrane-bound formate hydrogenlyase complex from *Escherichia coli*

**DOI:** 10.1038/s41467-022-34914-1

**Published:** 2022-11-25

**Authors:** Ralf Steinhilper, Gabriele Höff, Johann Heider, Bonnie J. Murphy

**Affiliations:** 1 Redox and Metalloprotein Research Group, Max Planck Institute of Biophysics, 60438 Frankfurt am Main, Germany; 2 Department of Biology, Laboratory for Microbial Biochemistry, Philipps University Marburg, 35043 Marburg, Germany; 3 Synmikro-Center for Synthetic Microbiology, Philipps University Marburg, 35043 Marburg, Germany

**Keywords:** Cryoelectron microscopy, Bacterial structural biology, Bioenergetics, Enzyme mechanisms

Correction to: *Nature Communications* 10.1038/s41467-022-32831-x, published online 14 September 2022

The original version of this Article contained an error in Fig. 5d, in which 3 columns of conserved residues were inadvertently hidden behind red markings instead of highlighted in red. This has been corrected in both the PDF and HTML versions of the Article.

