# Supplementary material for: Structure of the membrane-bound formate hydrogenlyase complex from Escherichia coli
Source: Nat Commun. 2022 Sep 14;13:5395. doi: 10.1038/s41467-022-32831-x (PMC9474812; doi:10.1038/s41467-022-32831-x)
Supplement: Supplementary file 1 — Supplementary Information [file 41467_2022_32831_MOESM1_ESM.pdf]

Supplementary Information for

Structure of the membrane-bound formate hydrogenlyase complex from *Escherichia coli*

Ralf Steinhilper[1], Gabriele Höff[2], Johann Heider[2,3], Bonnie J. Murphy[1*]

[1] Redox and Metalloprotein Research Group, Max Planck Institute of Biophysics, 60438 Frankfurt am Main, Germany

[2] Department of Biology, Laboratory for Microbial Biochemistry, Philipps University Marburg, 35043 Marburg, Germany

[3] Synmikro-Center for Synthetic Microbiology, Philipps University Marburg, 35043 Marburg, Germany

[*] Corresponding author. E-mail: bonnie.murphy@biophys.mpg.de

The PDF file includes:

Supplementary Table 1 - 3

Supplementary Figure 1 - 14

Supplementary References

Supplementary Table 1 Cryo-EM data collection, refinement and model validation statistics.

	FHL anaerobic	FHL aerobic
Map EMDB ID	**EMD-14429**	**EMD-14430**
**Data collection**		
Microscope	Titan Krios G2	Titan Krios G2
Camera	Gatan K3 Bioquantum	Gatan K2 Summit
Voltage (kV)	300	300
Nominal magnification	105,000x	165,000x
Calibrated pixel size (Å)	0.831*	0.828
Dose (e^-/Å^2)	61	72
Number of frames per image	61	40
Defocus range (µm)	-2.5 to -1.0	-2.2 to -1.6
**Image processing**		
Motion correction software	MotionCor2	MotionCor2
CTF estimation software	CTFFIND4	CTFFIND4
Particle selection software	Topaz	crYOLO
Final micrographs (no.)	7,338	1,207
Initial particle images (no.)	686,602	202,046
Final particle images (no.)	300,386	90,459
Map sharpening B-Factor (Å^2)	-66.6	**
Final resolution (Å)	2.6	3.0 – 3.4**
Model PDB ID	**7Z0S**	**7Z0T**
**Refinement**		
Modeling software	Coot, Phenix	Coot, Phenix
Protein residues	2,026	2,690
Water	109	-
Ligands		
NI	1	1
6MO	-	1
FE	1	1
MGD	-	2
SEC	-	1
SF4	7	8
FCO	1	1
DR9	1	-
PTY	2	-
CDL	1	-
LMN	1	-
**Validation**		
MolProbity score	1.77	1.95
Clash score	7.17	8.79
Ramachandran plot (%)		
Outliers	0.10	0.30
Allowed	2.68	4.42
Favored	97.22	95.28
Rotamer outliers (%)	2.00	1.55
Cβ outliers (%)	0.00	1.46
Peptide plane (%)		
Cis proline/general	6.2/0.0	5.0/0.0
Twisted proline/general	0.0/0.0	0.0/0.0
CaBLAM outliers (%)	1.30	2.64

* During refinement a pixel size of 0.837 Å was used. The final map was postprocessed using the calibrated pixel size of 0.831 Å.
** EMD-14430 is a composite map. The consensus and focused maps that contributed to the composite map are listed in Supplementary Table 2.

Supplementary Table 2 Cryo-EM data collection and refinement statistics for maps that contributed to the composite map EMD-14430.

	**EMD-14431**	**EMD-14432**	**EMD-14433**	**EMD-14434**
**Data collection**				
Microscope	Titan Krios G2	Titan Krios G2	Titan Krios G2	Titan Krios G2
Camera	Gatan K2 Summit	Gatan K2 Summit	Gatan K2 Summit	Gatan K2 Summit
Voltage (kV)	300	300	300	300
Nominal magnification	165,000x	165,000x	165,000x	165,000x
Calibrated pixel size (Å)	0.828	0.828	0.828	0.828
Dose ($e^-/Å^2$)	72	72	72	72
Number of frames per image	40	40	40	40
Defocus range (μm)	-2.2 to -1.6	-2.2 to -1.6	-2.2 to -1.6	-2.2 to -1.6
**Image processing**				
Motion correction software	MotionCor2	MotionCor2	MotionCor2	MotionCor2
CTF estimation software	CTFFIND4	CTFFIND4	CTFFIND4	CTFFIND4
Particle selection software	crYOLO	crYOLO	crYOLO	crYOLO
Final micrographs (no.)	1,207	1,207	1,207	1,207
Initial particle images (no.)	202,046	202,046	202,046	202,046
Final particle images (no.)	90,459	90,459	90,459	90,459
Map sharpening B-Factor ($Å^2$)	-54.5	-48.0	-43.7	-74.2
Final resolution (Å)	3.4	3.1	3.0	3.4

Supplementary Table 3 Subunit homology in complex I, FHL, MBH and soluble [NiFe] hydrogenases.

	**Complex I** *H. sapiens / Y. lipolytica*	*T. thermophilus*	**FHL** *E. coli*	**MBH** *P. furiosus*	**[NiFe] hydrogenase** *D. vulgaris*
**substrate oxidation**	NDUFS1	Nqo3	FdhF (C-term)<br>HycB (N-term)		
	NDUFV1	Nqo1			
	NDUFV2	Nqo2			
**substrate reduction**	NDUFS2	Nqo4	HycE (C-term)	MbhL	HydB
	NDUFS3	Nqo5	HycE (N-term)	MbhK	
	NDUFS7	Nqo6	HycG	MbhJ	HydA
	NDUFS8	Nqo9	HycF	MbhN	
**(putative) proton trans-location**	ND1	Nqo8	HycD	MbhM	
	ND2/ND4/ND5	Nqo14/Nqo13/Nqo12	HycC (N-term)	MbhH	
	ND3	Nqo7	HycC (C-term)	MbhI (N-term)	
	ND6	Nqo10		MbhD/MbhE	
	ND4L	Nqo11		MbhG	
**sodium trans-location**				MbhF	
				MbhA	
				MbhB	
				MbhC	

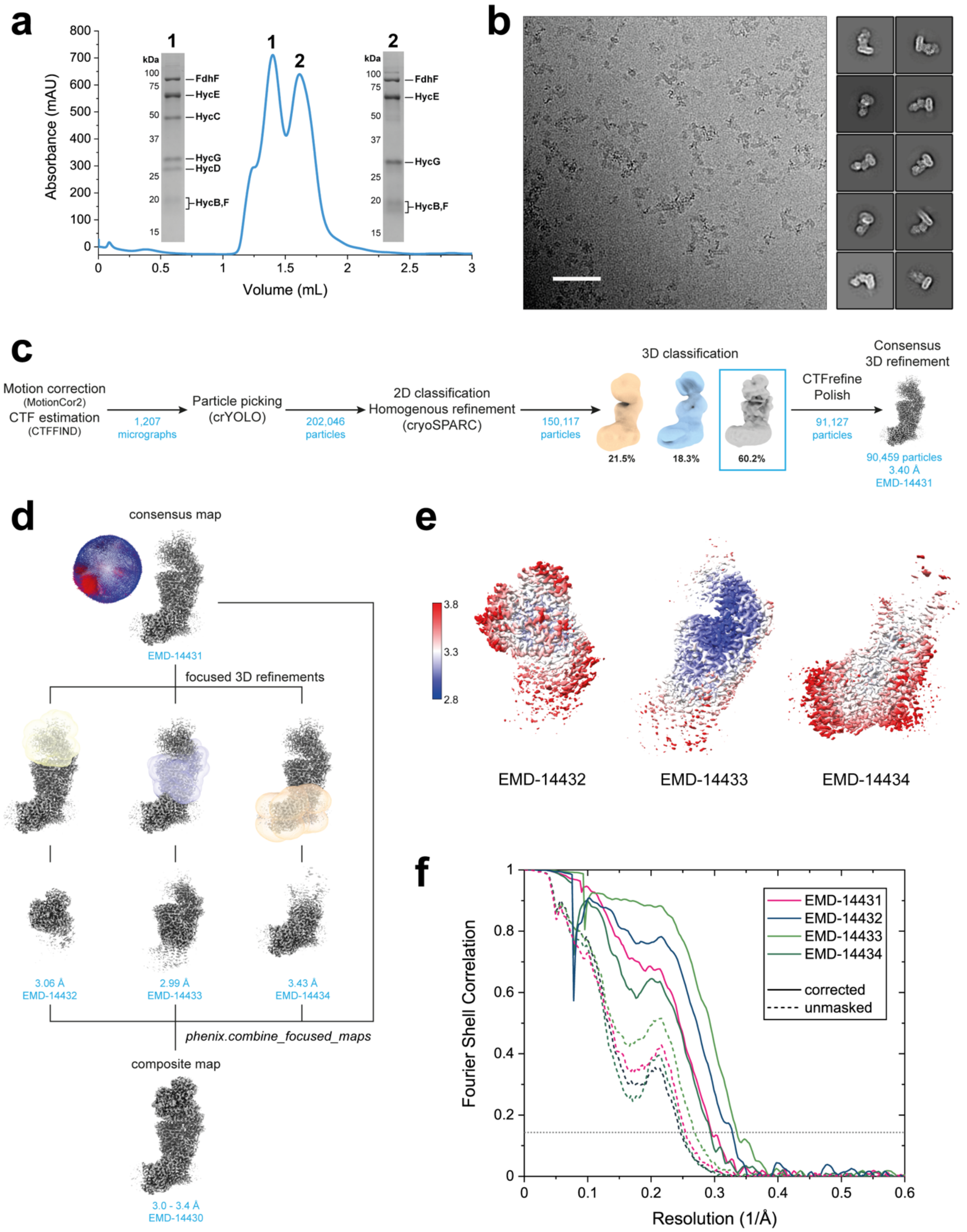

Supplementary Figure 1 Sample preparation and data processing of aerobically prepared FHL. (**a**) Representative size exclusion chromatography (SEC) profile (1 out of 2) performed on a Superdex 200 Increase 5/150 GL column and SDS-PAGE of peak fractions corresponding to peak 1 (entire heptameric complex) and peak 2 (soluble arm subunits). Gel bands are labeled according to molecular mass. Source data are provided as a Source Data file. (**b**) Representative cryo-EM micrograph (1 out of 1,207), scale bar represents 500 Å. Corresponding 2D class averages. (**c**) Processing workflow, with all steps performed in RELION-3 except where otherwise indicated. A consensus 3D refinement yields a map (EMD-14431) with a resolution of 3.4 Å. (**d**) The consensus map served as a basis for masked focused 3D refinement, giving focused maps of all regions of the complex (EMD-14432 – EMD-14434) with resolutions between 3.0 - 3.4 Å, which were combined using *phenix.combine_focused_maps*. The resulting composite map (EMD-14430) was used for model refinement. (**e**) Local resolution estimation for focused refinements EMD-14432 – 14434. (**f**) Fourier Shell Correlation (FSC) curves for EMD-14431 – 14434.

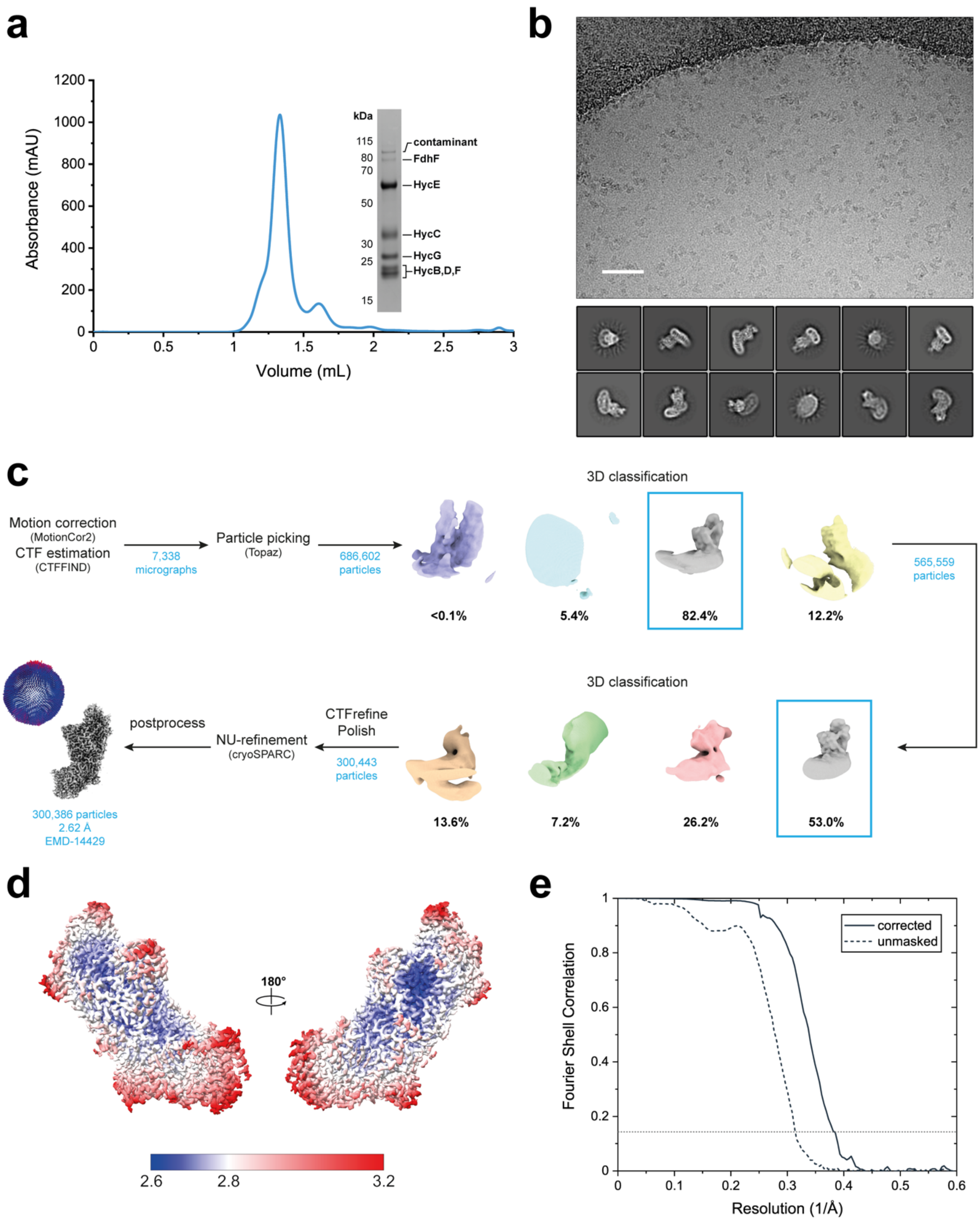

Supplementary Figure 2 Sample preparation and data processing of anaerobically prepared FHL. (**a**) Representative size exclusion chromatography (SEC) profile (1 out of 2) on a Superdex 200 Increase 5/150 GL column and SDS-PAGE. Gel bands are labeled according to molecular mass. Source data are provided as a Source Data file. Traces of a ~80 kDa band could be identified in the gel, but no density for FdhF was observed in any 2D class averages or 3D reconstructions of the cryo-EM dataset. A higher molecular weight band is a putative contaminant. (**b**) Representative cryo-EM micrograph (1 out of 7,338), scale bar represents 500 Å. Corresponding 2D class averages. (**c**) Processing workflow, with all processing steps performed in RELION-3.1 except where otherwise indicated. The angular distribution plot was generated from the non-uniform refinement using pyem[1]. (**d**) Local resolution estimation of the final postprocessed map EMD-14429. (**e**) Fourier Shell Correlation (FSC) curves of the final postprocessed map EMD-14429.

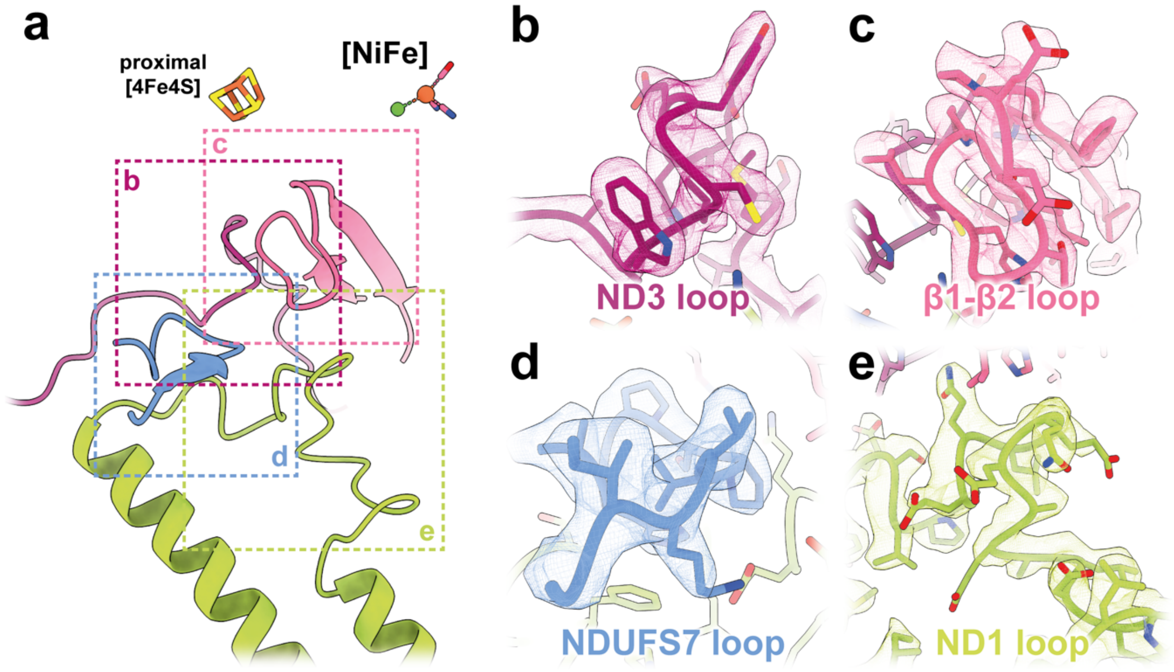

Supplementary Figure 3 Conserved loop cluster. (**a**) Four peptide loops in the hydrogenase subunits (HycE and HycG) and membrane subunits (HycD and HycC) form a conserved loop cluster at the interface of the soluble and membrane arms. Our cryo-EM map of anaerobically prepared FHL shows side chain density for (**b**) the ND3 loop region between TMH15 and TMH16 of HycC, (**c**) the β1-β2 loop in the large hydrogenase subunit HycE, (**d**) the NDUFS7 loop in the small hydrogenase subunit HycG and (**e**) the ND1 loop between TMH5 and THM6 of HycD.

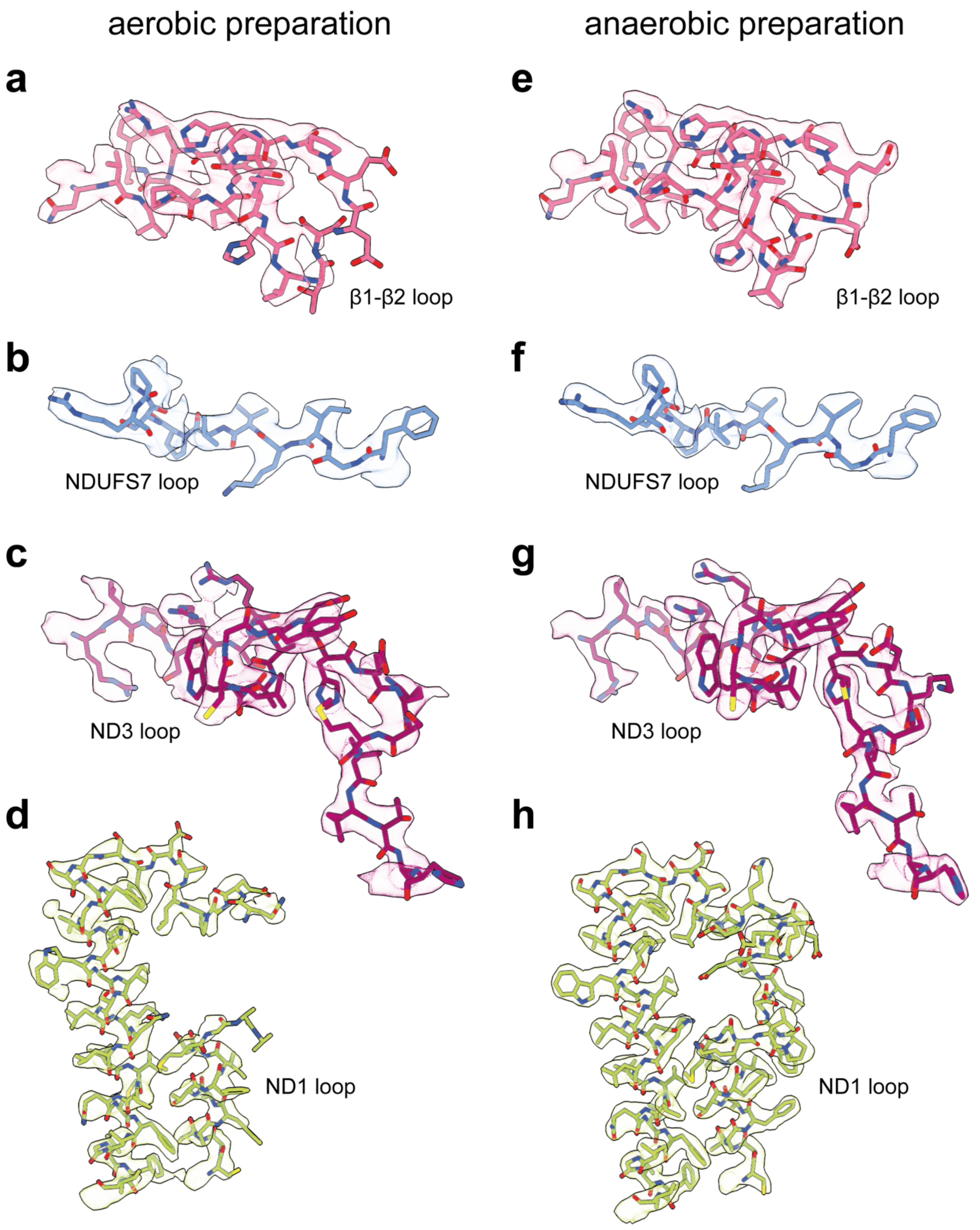

Supplementary Figure 4 Comparison of aerobically and anaerobically-purified FHL in the loop region connecting the membrane and soluble arms. Model and cryo-EM density for the conserved loop cluster in aerobically (**a-d**) and anaerobically (**e-h**) prepared FHL. The ND1 loop is partially unresolved for the aerobically prepared sample, whereas the anaerobically prepared sample shows side chain density for all loops.

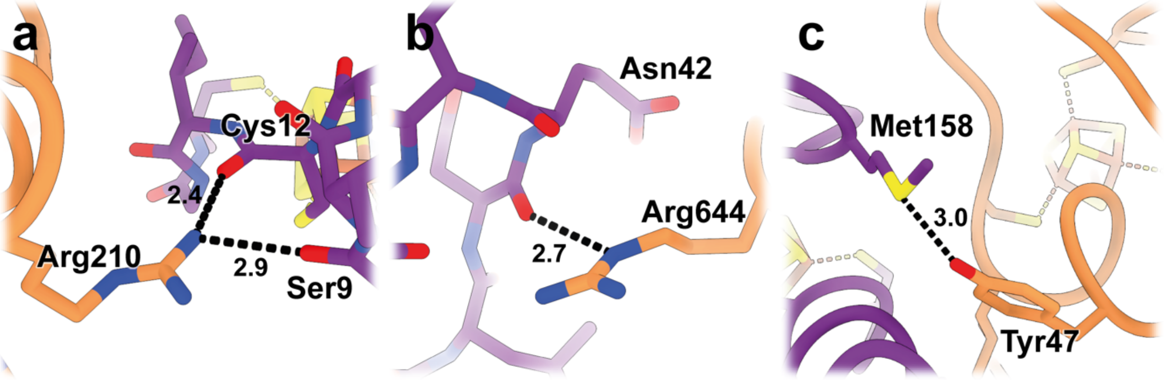

Supplementary Figure 5 Regions of hydrogen-bonding between FdhF and HycB (**a**) Arg210 of FdhF interacts with backbone oxygens of Cys12 and Ser9 of HycB (**b**) Arg644 of FdhF interacts with backbone oxygen of Asn42 of HycB (**c**) Tyr47 of FdhF interacts with Met158 of HycB.

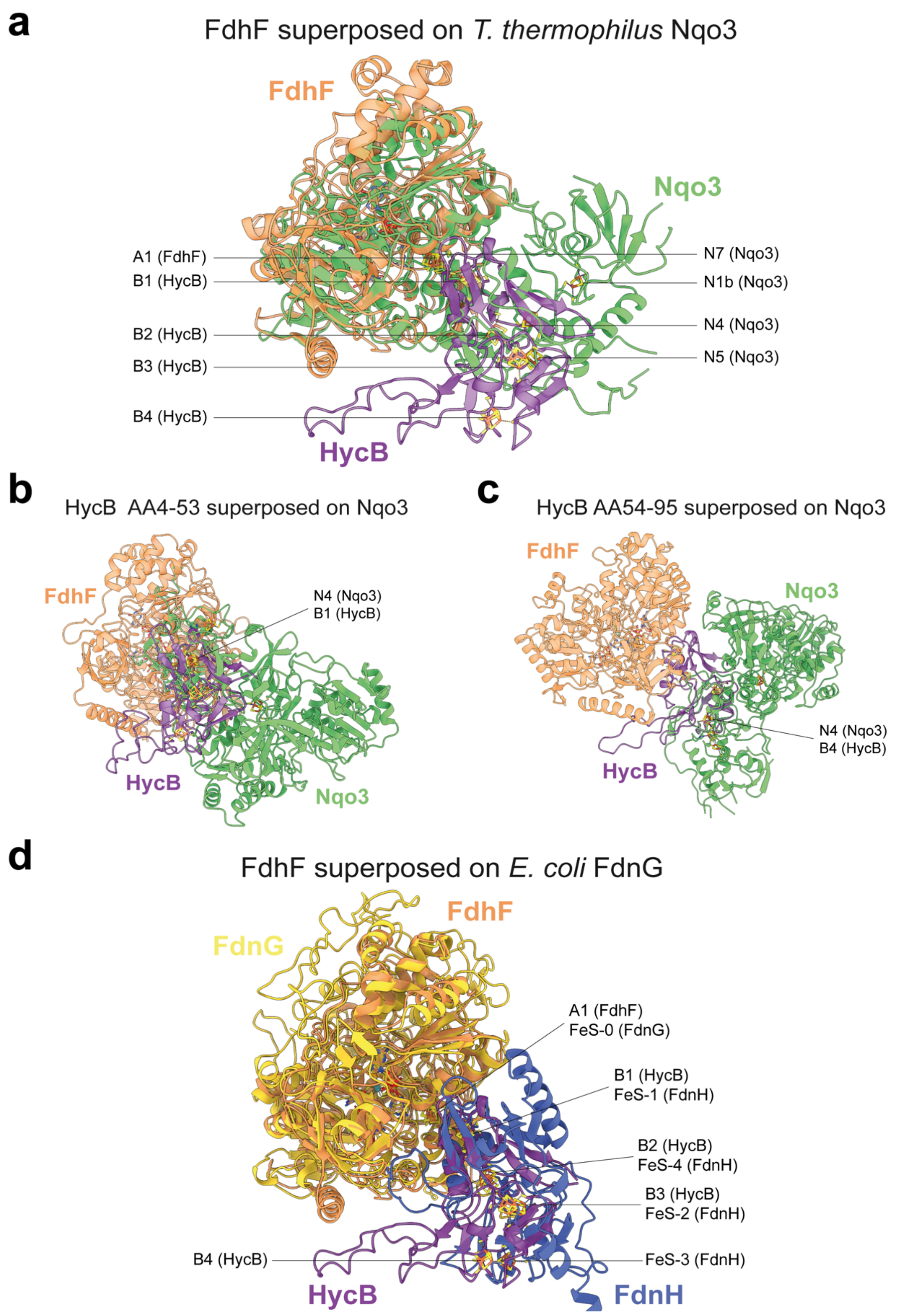

Supplementary Figure 6 Superposition of FdhF with homologues. (**a**) *E. coli* FdhF can be superposed with the C-terminal region of *T. thermophilus* complex I Nqo3 (PDB 4HEA[2]) with an RMSD of 6.610 Å across all 496 atom pairs, bringing the [4Fe4S] cluster A1 in FdhF in register with the off-pathway [4Fe4S] cluster N7 in Nqo3. (**b**) HycB residues 4-53 can be superposed on the N-terminal region of Nqo3 with an RMSD of 10.306 Å over all 48 atom pairs, so that the [4Fe4S] cluster B1 (HycB) is in register with N4 (Nqo3), but in this orientation the FdhF subunit is not aligned with Nqo3. (**c**) HycB residues 54-95 can be superposed on the N-terminal region of Nqo3 with an RMSD of 14.609 Å across all 38 atom pairs, so that the [4Fe4S] cluster B4 (HycB) matches N4 (Nqo3), but the FdhF subunit does not match Nqo3. (**d**) FdhF superposed on the crystal structure of the *E. coli* formate dehydrogenase-N (PDB: 1KQF[3]) with an RMSD of 8.370 Å over 677 atom pairs, closely resembles the quarternary structure of the FdhF-HycB interaction. All [4Fe4S] clusters in FdhF and HycB of *E. coli* FHL are in register with those of *E. coli* Fdh-N.

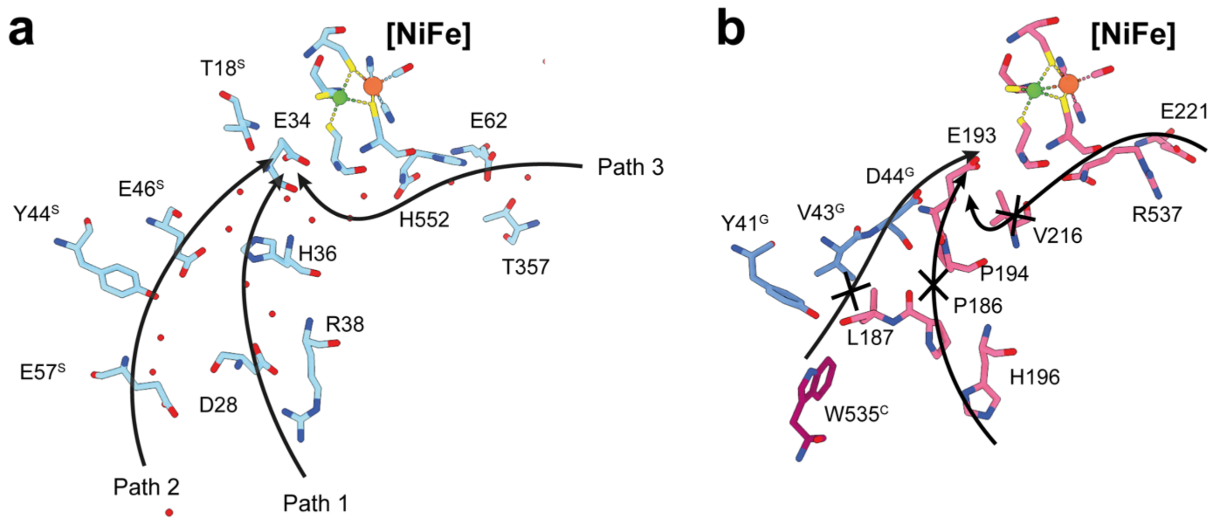

Supplementary Figure 7 Substrate proton pathways in a soluble [NiFe] hydrogenase from *D. vulgaris* and Hyd-3 of FHL. (**a**) Proton pathways observed in the crystal structure of *D. vulgaris* [NiFe] hydrogenase (PDB 4U9H[4]). Proton paths 1-3 leading to Glu34 as discussed in[4] are shown with arrows. (**b**) The residues forming these pathways are not conserved in *E. coli* FHL; in many cases they have been replaced by hydrophobic residues that appear to block proton transfer.

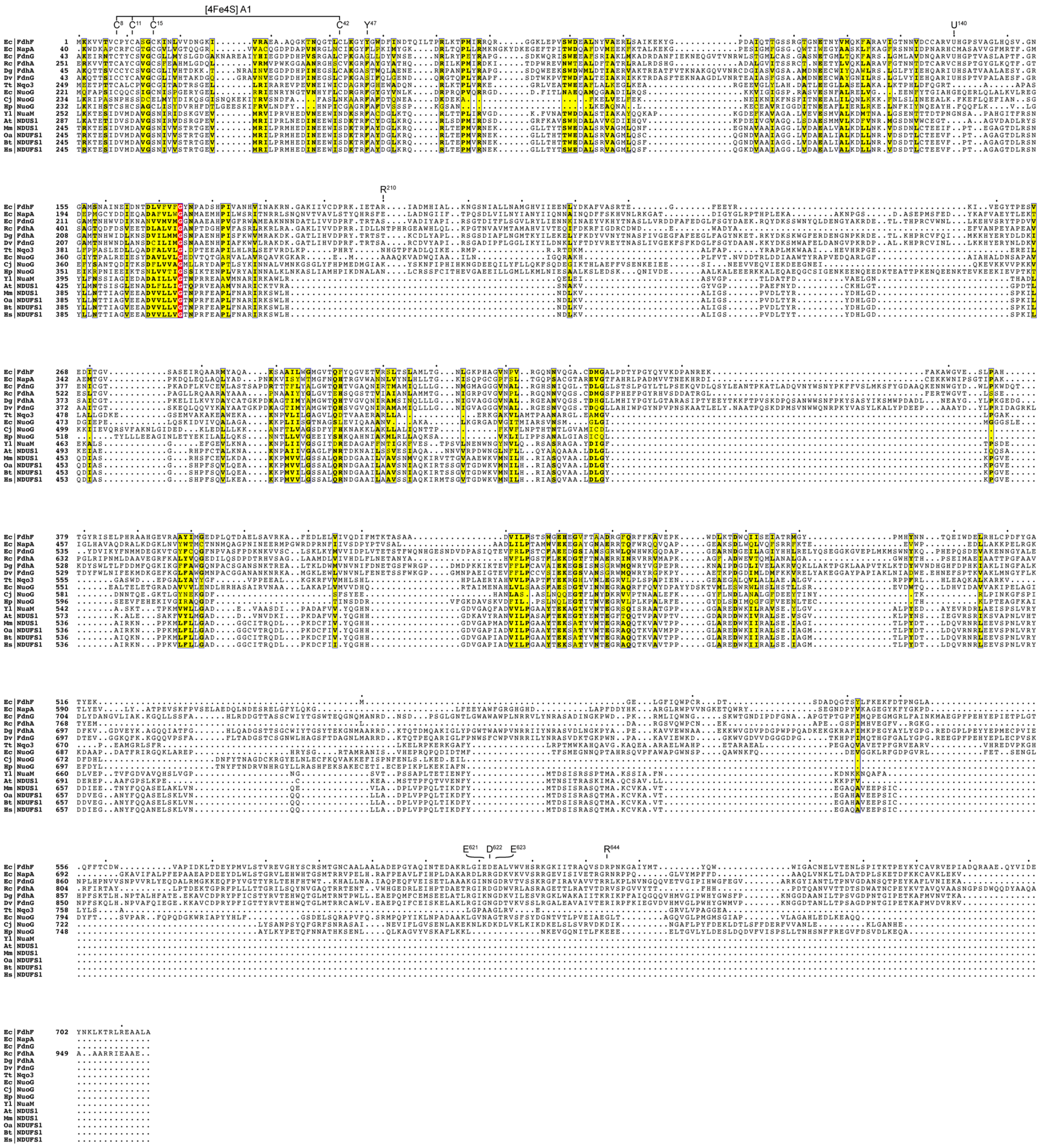

Supplementary Figure 8 Sequence alignment of the formate dehydrogenase H (FdhF). *Escherichia coli* formate dehydrogenase H (Ec|FdhF: P07658) was aligned with nitrate reductase from *E. coli* (Ec|NapA: P33937) and with formate dehydrogenases from *E. coli* (Ec|FdnG: P24183), *Rhodobacter capsulatus (*Rc|FdhA: D5AQH0), *Desulfovibrio gigas* (Dg|FdhA: Q934F5), and *Desulfovibrio vulgaris* (Dv|FdnG: Q72EJ1) as well as with the C-terminus of complex I subunit NDUFS1 from various species: *Thermus thermophilus* (Tt|Nqo3: Q56223), *E. coli* (Ec|NuoG: P33602), *Campylobacter jejuni* (Cj|NuoG: Q0P855), *Helicobacter pylori* (Hp|NuoG: I9X510), *Yarrowia lipolytica* (Yl|NuaM: Q9UUU3), *Arabidopsis thaliana* (At|NDUS1: Q9FGI6), *Mus musculus* (Mm|NDUFS1: Q91VD9), *Ovis aries* (Oa|NDUFS1: W5QB34), *Bos taurus* (Bt|NDUFS1: P15690), *Homo sapiens* (Hs|NDUFS1: P28331). The selenocysteine (U140) and cysteines coordinating cluster A1 in FdhF are marked (C8, C11, C15, C42). Residues interacting with HycB (Y47, R210, R544) and with HycF (E621, D622, E623) are marked.

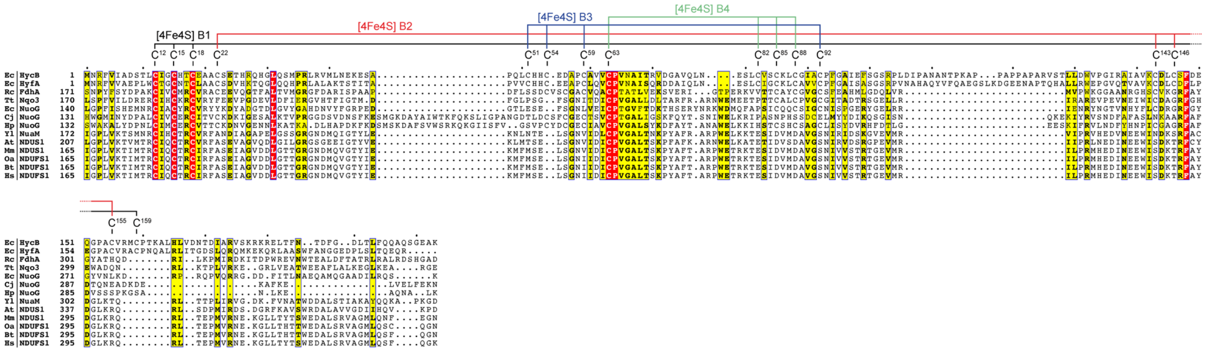

Supplementary Figure 9 Sequence alignment of HycB. *Escherichia coli* HycB (Ec|HycB: P0AAK1) was aligned with its homologues in *E. coli* FHL-2 (Ec|HyfA: P23481), *Rhodobacter capsulatus* (Rc|FdhA: D5AQH0) and the N-terminus of complex I subunit NDUFS1 from various species: *Thermus thermophilus* (Tt|Nqo3: Q56223), *E. coli* (Ec|NuoG: P33602), *Campylobacter jejuni* (Cj|NuoG: Q0P855), *Helicobacter pylori* (Hp|NuoG: I9X510), *Yarrowia lipolytica* (Yl|NuaM: Q9UUU3), *Arabidopsis thaliana* (At|NDUFS1: Q9FGI6), *Mus musculus* (Mm|NDUFS1: Q91VD9), *Ovis aries* (Oa|NDUFS1: W5QB34), *Bos taurus* (Bt|NDUFS1: P15690), *Homo sapiens* (Hs|NDUFS1: P28331). Cysteines ligating cluster B1 (C12, C15, C18, C159), B2 (C22, C143, C146, C155), B3 (C51, C54, C59, C92) and B4 (C63, C82, C85, C88) are marked.

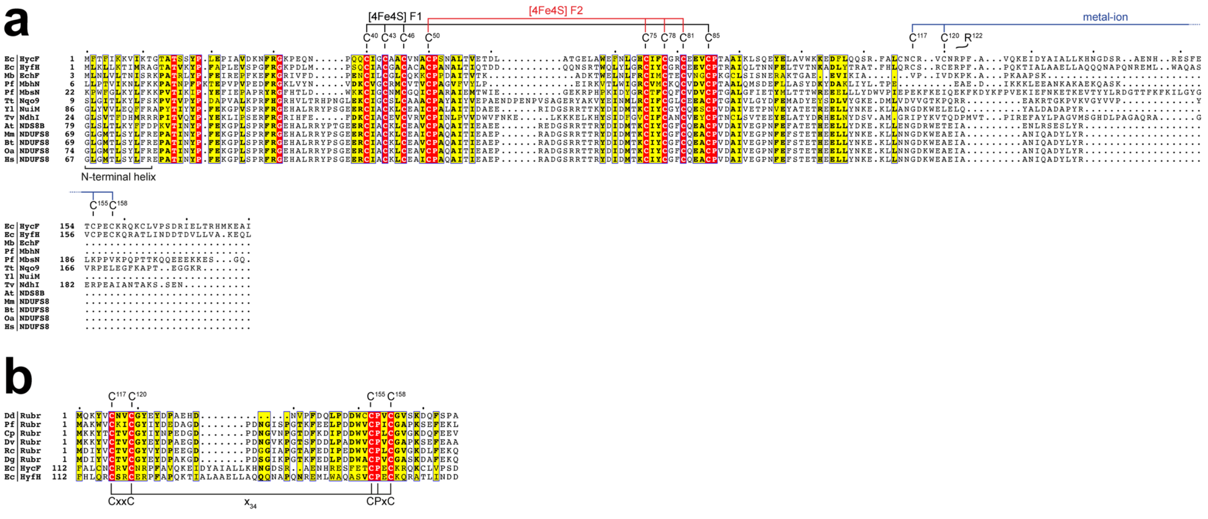

Supplementary Figure 10 Sequence alignment of HycF. (**a**) *Escherichia coli* HycF (Ec|HycF: P16432) was aligned with its homologues in the complex I superfamily: *E. coli* (Ec|HyfH: P77423), *Methanosarcina barkeri* (Mb|EchF: O59657), *Pyrococcus furiosus* (Pf|MbhN: I6U851), *P. furiosus* (Pf|MbsN: I6U853), *Thermus thermophilus* (Tt|Nqo9: Q56224), *Yarrowia lipolytica* (Yl|NuiM: Q9UUT8), *Thermosynechococcus elongatus* (Tv|NdhI: Q8DL31), *Arabidopsis thaliana* (At|NDS8B: Q9FX83), *Mus musculus* (Mm|NDUFS8: Q8K3J1), *Bos taurus* (Bt|NDUFS8: P42028), *Ovis aries* (Oa|NDUFS8: A0A7M4DUG4), *Homo sapiens* (Hs|NDUFS8: O00217). Cysteines ligating cluster F1 (C40, C43, C46, C85) and cluster F2 (C50, C75, C78, C81) and cysteines coordinating the unpredicted metal ion (C117, C120, C155, C158) as well as R122, which interacts with FdhF, are marked. The N-terminal helix of HycF is a common feature in the complex I superfamily. (**b**) The unexpected ion-binding site of *E. coli* HycF (Ec|HycF: P16432) is aligned with homologues from *E. coli* FHL-2 (Ec|HyfH: P77423) and rubredoxins from various species: *Desulfovibrio desulfuricans* (Dd|Rubr: P04170), *Pyrococcus furiosus* (Pf|Rubr: P24297), *Clostridium pasteurianum* (Cp|Rubr: P00268), *Desulfovibrio vulgaris* (Dv|Rubr: P00269), *Ruminiclostridium cellulolyticum* (Rc|Rubr: Q9X709), *Desulfovibrio gigas* (Dg|Rubr: P00270). The unpredicted metal ion-binding motif (CxxC-x_{34}-CPxC) of HycF is marked.

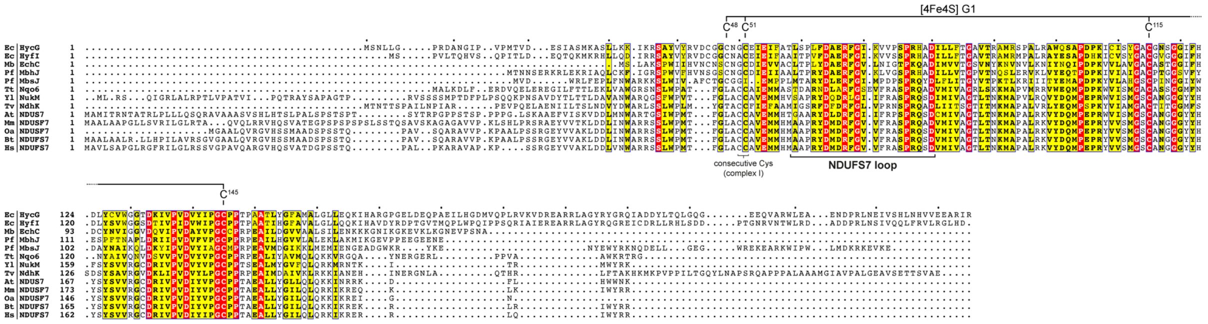

Supplementary Figure 11 Sequence alignment of HycG. *Escherichia coli* HycG (Ec|HycG: P16433) was aligned with its homologues in the complex I superfamily: hydrogenases from *E. coli* (Ec|HyfI: P77668), *Methanosarcina barkeri* (Mb|EchC: O59654), *Pyrococcus furiosus* (Pf|MbhJ: Q8U0Z8), the MBS complex from *P. furiosus* (Pf|MbsJ: I6UZV3), and complex I from *Thermus thermophilus* (Tt|Nqo6: Q56218), *Yarrowia lipolytica* (Yl|NukM: Q9UUT7), *Thermosynechococcus elongatus* (Tv|NdhK: Q8DKZ4), *Arabidopsis thaliana* (At|NDUS7: Q42577), *Mus musculus* (Mm|NDUSF7: Q9DC70), *Ovis aries* (Oa|NDUSF7: W5PPP6), *Bos taurus* (Bt|NDUFS7: P42026), and *Homo sapiens* (Hs|NDUFS7: O75251). Cysteines ligating the proximal cluster G1 (C45, C51, C115, C145) and the NDUFS7 loop region are marked.

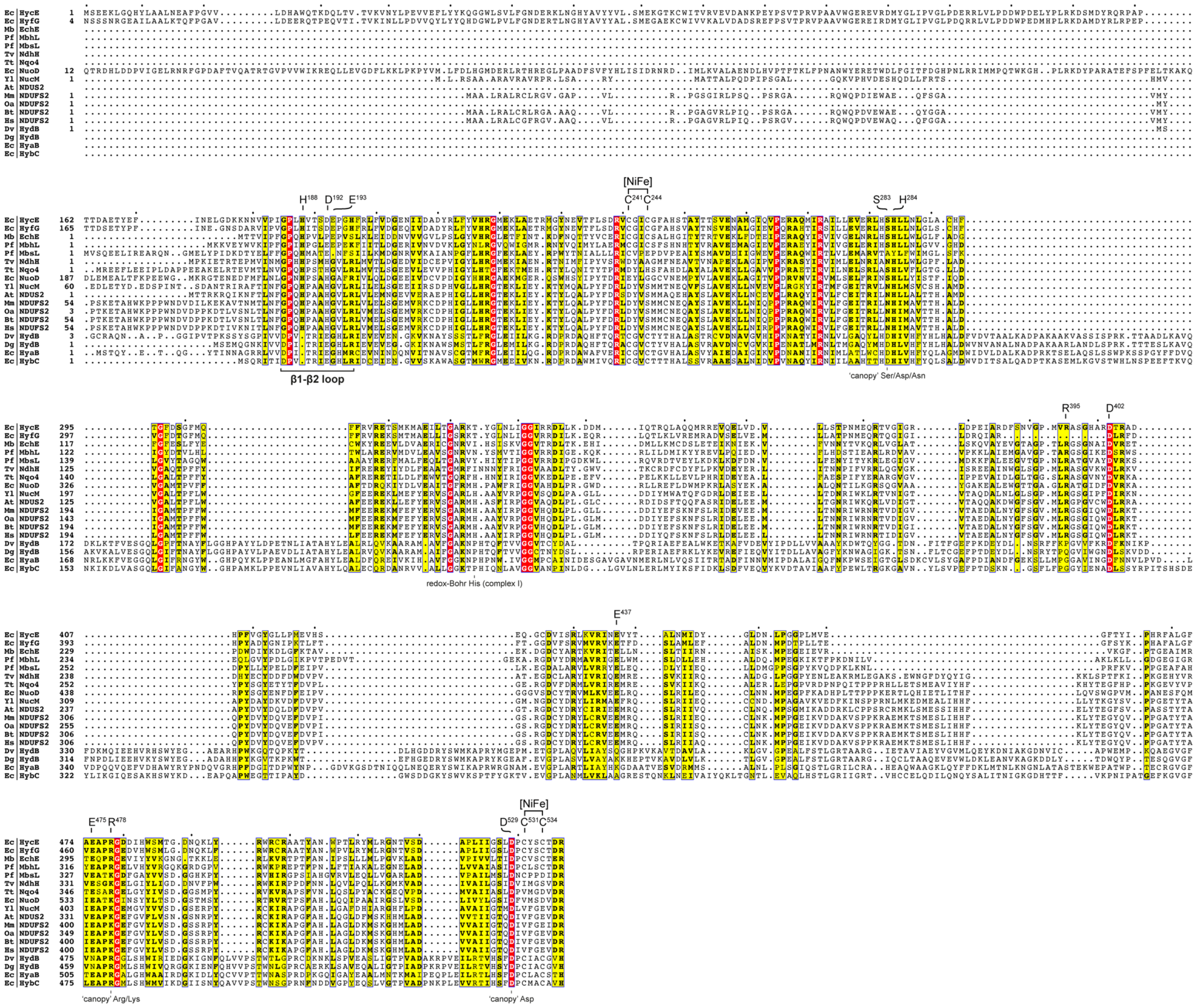

Supplementary Figure 12 Sequence alignment of HycE. *Escherichia coli* Hyd-3 large subunit (Ec|HycE: P16431) was aligned with its homologues in the complex I superfamily: *E. coli* (Ec|HyfG: P77329), *Methanosarcina barkeri* (Mb|EchE: O59656), *Pyrococcus furiosus* (Pf|MbhL: Q8U0Z6), *P. furiosus* (Pf|MbsL: I6V297), *Thermosynechococcus elongatus* (Tv|NdhH: Q8DJD9), *Thermus thermophilus* (Tt|Nqo4: Q56220), *E. coli* (Ec|NuoD: P33599), *Yarrowia lipolytica* (Yl|NucM: Q9UUU1), *Arabidopsis thaliana* (At|NDUS2: P93306), *Mus musculus* (Mm|NDUFS2: Q91WD5), *Ovis aries* (Oa|NDUFS2: W5PJ73), *Bos taurus* (Bt|NDUFS2: P17694), *Homo sapiens* (Hs|NDUFS2: O75306), and with [NiFe] hydrogenases from *Desulfovibrio vulgaris* (Dv|HydB: P21852), *Desulfovibrio gigas,* (Dg|HydB: P12944) as well as *E. coli* Hyd-1 (Ec|HyaB: P0ACD8) and *E. coli* Hyd-2 (Ec|HybC: P0ACE0). Cysteines (C241, C244, C531, C534) coordinating the [NiFe] cofactor, [NiFe] site 'canopy' residues (S283, R478, D529), residues of the putative substrate proton pathway (H284, R395, D402, E437, E475) and residues of the β1-β2 loop region (D192, E193) are marked. The alignment represents the protein sequences after proteolytic maturation.

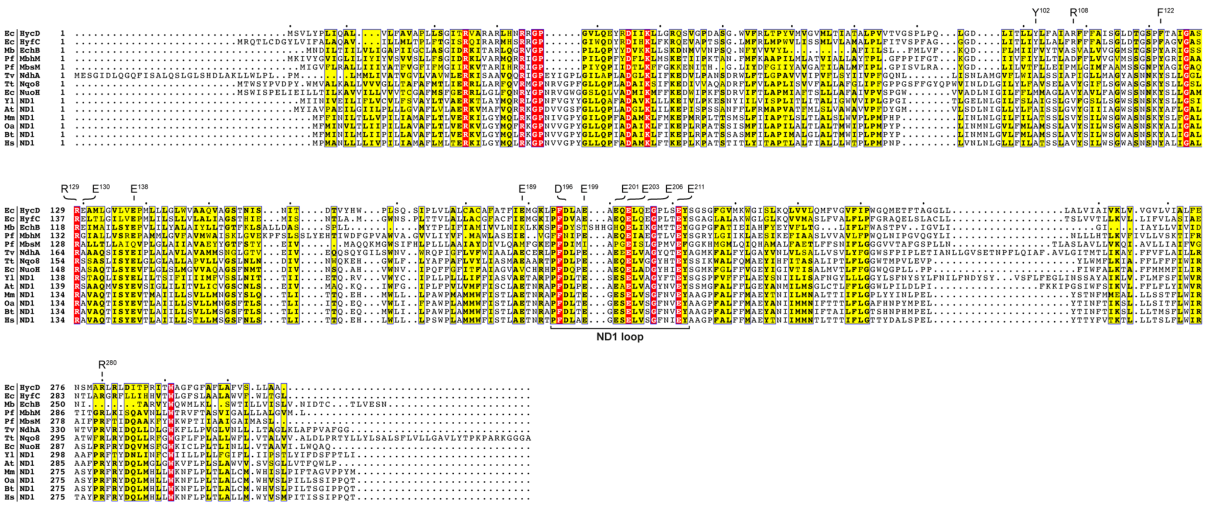

Supplementary Figure 13 Sequence alignment of HycD. *Escherichia coli* HycD (Ec|HycD: P16430) was aligned with its homologues in the complex I superfamily: *E. coli* (Ec|HyfC: P77858), *Methanosarcina barkeri* (Mb|EchB: O59653), *Pyrococcus furiosus* (Pf|MbhM: I6UQM0), *P. furiosus* (Pf|MbsM: I6V2A1), *Thermosynechococcus elongatus* (Tv|NdhA: Q8DL32),*Thermus thermophilus* (Tt|Nqo8: Q60019), *E. coli* (Ec|NuoH: P0AFD4), *Yarrowia lipolytica* (Yl|ND1: Q9B6E8), *Arabidopsis thaliana* (At|ND1: P92558), *Mus musculus* (Mm|ND1: P03888), *Ovis aries* (Oa|ND1: O78747), *Bos taurus* (Bt|ND1: P03887), *Homo sapiens* (Hs|ND1: P03886). Residues of the E-channel as well as the ND1 loop are marked.

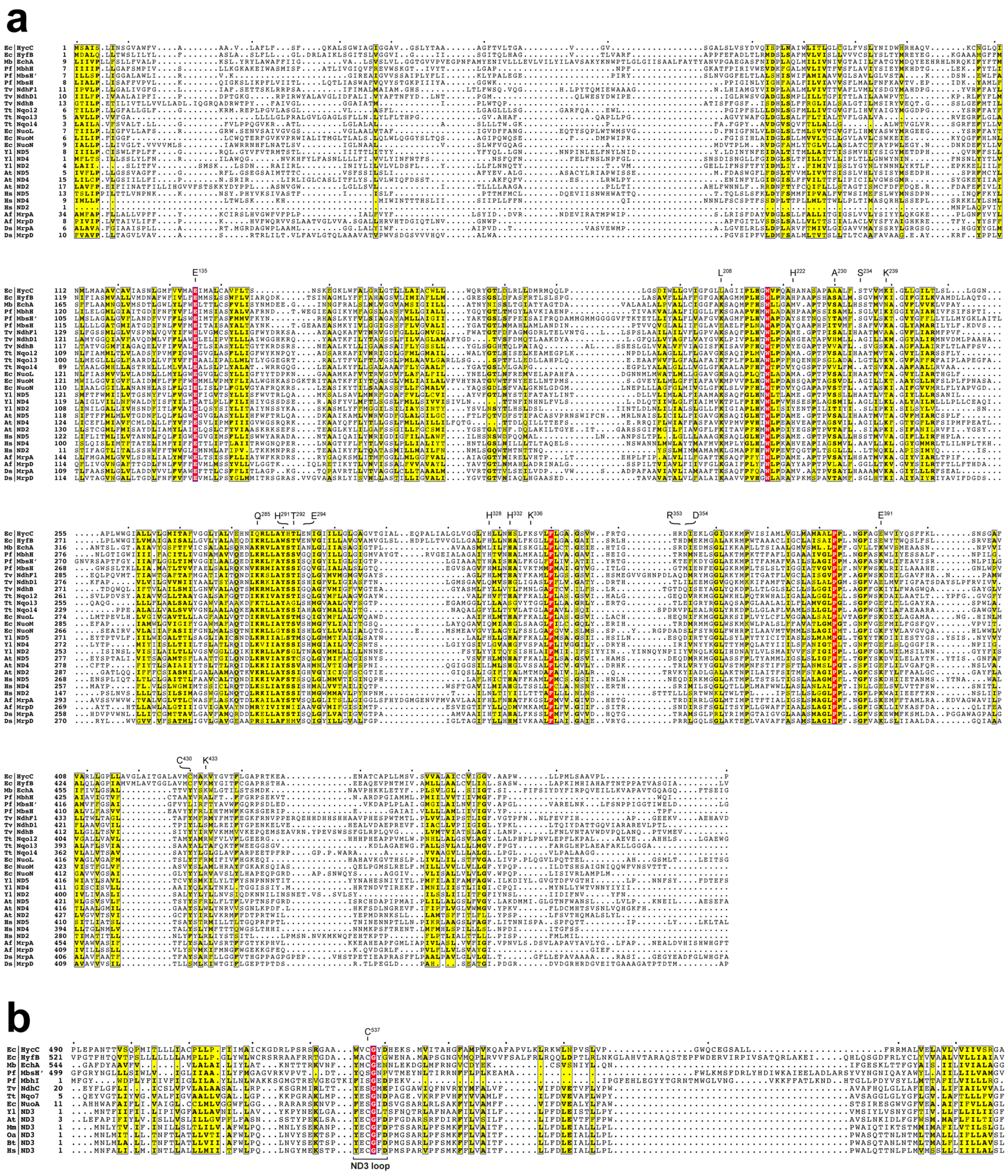

Supplementary Figure 14 Sequence alignment of HycC. (**a**) Alignment of *Escherichia coli* HycC (Ec|HycC: P16429) residues 1-490 with antiporter-like subunits in the complex I superfamily: *E. coli* (Ec|HyfB: P23482), *Methanosarcina barkeri* (Mb|EchA: O59652), *Pyrococcus furiosus* (Pf|MbhH: I6UQL5, Pf|MbsH': I6TXQ1, Pf|MbsH: I6UZV7), *Thermosynechococcus elongatus* (Tv|NdhF1: Q8DKX9, Tv|NdhD1: Q8DKY0, Tv|NdhB: Q8DMR6), *Thermus thermophilus* (Tt|Nqo12: Q56227, Tt|Nqo13: Q56228, Tt|Nqo14: Q56229), *E. coli* (Ec|NuoL: P33607, Ec|NuoM: P0AFE8, Ec|NuoN: P0AFF0), *Yarrowia lipolytica* (Yl|ND5: Q9B6D3, Yl|ND4: Q9B6D6, Yl|ND2: Q9B6C8), *Arabidopsis thaliana* (At|ND5: P29388, At|ND4: P93313, At|ND2: O05000), *Homo sapiens* (Hs|ND5: P03915, Hs|ND4: P03905, Hs|ND2: P03891), and with antiporter-like subunits of MRP: *Anoxybacillus flavithermus* (Af|MrpA: B7GL84, Af|MrpD: B7GL98), *Dietzia sp.* (Ds|MrpA: A0A221C8X2, Ds|MrpD: A0A221C8X0). Residues referred to in Figure 5c are marked. (**b**) Alignment of *E. coli* HycC residues 490-608 with the C-terminal part of related antiporter-like subunits in the complex I superfamily and with complex I subunit ND3 from various species: *P. furiosus* (Pf|MbhI: I6U847), *T. elongatus* (Tv|NdhC: Q8DJ02), *T. thermophilus* (Tt|Nqo7: Q56217), *E. coli* (Ec|NuoA: P0AFC3), *Y. lipolytica* (Yl|ND3: Q9B6C7), *A. thaliana* (At|ND3: P92533), *M. musculus* (Mm|ND3: P03899), *Ovis aries* (Oa|ND3: O78753), *Bos taurus* (Bt|ND3: P03898), *Homo sapiens* (Hs|ND3: P03897).

Supplementary References

1. Asarnow, D., Palovcak, E. & Cheng, Y. asarnow/pyem: UCSF pyem v0.5. *Zenodo* (2019). doi:10.5281/zenodo.3576630
2. Baradaran, R., Berrisford, J. M., Minhas, G. S. & Sazanov, L. A. Crystal structure of the entire respiratory complex I. *Nature* **494,** 443–448 (2013).
3. Jormakka, M., Törnroth, S., Byrne, B. & Iwata, S. Molecular basis of proton motive force generation: structure of formate dehydrogenase-N. *Science* **295,** 1863–1868 (2002).
4. Ogata, H., Nishikawa, K. & Lubitz, W. Hydrogens detected by subatomic resolution protein crystallography in a [NiFe] hydrogenase. *Nature* **520,** 571–574 (2015).
